# Real-time Polymerase Chain Reaction To Diagnose Lymphogranuloma Venereum

**DOI:** 10.3201/eid1108.050535

**Published:** 2005-08

**Authors:** Servaas A. Morré, Joke Spaargaren, Johannes S.A. Fennema, Henry J.C. de Vries, Roel A. Coutinho, A. Salvador Peña

**Affiliations:** * VU University Medical Center, Amsterdam, the Netherlands;; † Municipal Health Service, Amsterdam, the Netherlands;; ‡ Academic Medical Center, Amsterdam, the Netherlands;; § National Institute for Public Health and the Environment, Bilthoven, the Netherlands

**Keywords:** LGV, real time PCR, Chlamydia trachomatis

**To the Editor:** An outbreak of rectal lymphogranuloma venereum (LGV) has been detected in the Netherlands among men who have sex with men (1–4). More cases of LGV in other European countries such as Belgium, France, and the United Kingdom have been reported, and the first cases have been detected in the United States as well. This infection is encountered not only by clinicians who treat sexually transmitted diseases but also by gastroenterologists. Both the European Surveillance of Sexually Transmitted Infections (http://www.essti.org) and the Centers for Disease Control and Prevention (http://www.cdc.gov) are working on outbreak warning and response systems to increase the awareness and the direct management of the LGV outbreak (5,6).

Different approaches have been described to diagnose LGV infections (Figure). The first 3 approaches have serious disadvantages: cell culture is rarely available in routine diagnostic settings, polymerase chain reaction (PCR)-based restriction fragment length polymorphism (RFLP) analysis (usually nested PCR approaches are used) needs post-PCR restriction enzyme profiling, and sequencing requires additional analyses of sequence data to identify the *Chlamydia trachomatis* serovar responsible for infection. In addition, all 3 techniques are time consuming (at least 1–4 days to get a result), laborious, and require specially trained personnel in a sophisticated laboratory setting. Therefore, we developed a real-time PCR approach (TaqMan and Rotorgene) that can easily identify LGV strains in 2 hours with equipment that is available in almost all diagnostic settings.

**Figure:**
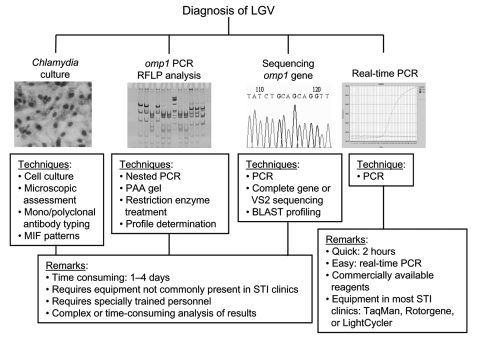
Diagnosis of lymphogranuloma venereum. MIF, microimmunofluorescence; STI, sexually transmitted infection; PCR, polymerase chain reaction; RFLP, restriction fragment length polymorphism; PAA, poly acrylamide; BLAST, basic local alignment search tool.

We used the polymorphic membrane protein H gene (*pmp* gene) as a PCR target because it has a unique gap in LGV strains of *C. trachomatis*, compared to other serovars, which makes it highly specific. The following primers and probes were selected: LGV-F 5´ CTG TGC CAA CCT CAT CAT CAA 3´, LGV-R 5´ AGA CCC TTT CCG AGC ATC ACT 3´, and LGV MGB-probe 6-FAM-CCT GCT CCA ACA GT. Real-time PCR conditions (20-μL format) for TaqMan were as follows: 2× TaqMan Universal Mastermix (Applied Biosystems, Foster City, CA, USA), 18 pmol each primer, 0.2 μmol/L probe, and 2 μL (LGV L2) DNA or clinical sample; 2 min at 50°C, 10 min at 95°C, and 40 cycles of 15 sec at 95°C and 1 min at 60°C. Conditions for Rotorgene were as follows: 10× buffer (Hoffman-La Roche Ltd, Basel, Switzerland), 10 pmol each primer, 0.04 μmol/L probe, 2 μL (LGV L2) DNA or clinical sample; 2 min at 50°C, 10 min at 95°C, and 45 cycles of 15 sec at 95°C and 1 min at 60°C. By using a previously described serial dilution of LGV L2 (7), sensitivity was assessed as 0.01 inclusion-forming units for both real-time PCR assays.

To determine specificity, we tested different *C. trachomatis* serovars and serovariants A, B, Ba, C, D, Da, D-, E, F, G, Ga, H, I, Ia, I-, J, Jv, K, L1, L2, L2b, L3, *C. muridarum* (MoPn), *C. pneumoniae*, *C. pecorum*, *C. psittaci*, and 32 other microorganisms that normally reside in the human perianal and urogenital region and in the oropharynx. These organisms included gram-positive and gram-negative bacteria and yeast: *Acinetobacter baumannii*, *Campylobacter jejuni*, *Candida albicans*, other yeast, *Enterobacter agglomerans*, *Enterococcus faecalis*, *Escherichia coli*, *Streptococcus* spp., *Haemophilus influenzae*, *Klebsiella pneumoniae*, *Mycoplasma* spp., *Neisseria meningitidis*, *Pasteurella* spp., *Pseudomonas aeruginosa*, *Salmonella enteritidis*, *Shigella sonnei*, *Staphylococcus aureus*, and others. Only LGV strains L1, L2, L2b, and L3 tested positive in both the TaqMan and Rotorgene assays, which shows the analytical specificity of real-time PCR.

Subsequently, we determined in a blinded setting the presence of LGV in a selected group of patients (clinical spectrum and epidemiology described elsewhere [8]) according to *C. trachomatis*–positive rectal swab (Chlamydia 2SP Collection & Transport Kit [Quelab] by commercially available PCR (COBAS AMPLICOR, Hoffman-La Roche Ltd). By using the 2 reference standard techniques to type *C. trachomatis* serovars (PCR-based RFLP of the *omp1* gene or sequencing the variable segment 2 [VS-2] of the *omp1* gene) (9,10) with DNA isolated from rectal swab specimens (standard isopropanol DNA isolation method), we identified 28 of 125 men as LGV-positive. These 28 samples were also positive in both the TaqMan and Rotorgene assays. We also identified 2 additional LGV infections, which were initially typed and then retested as single-strain infections with serovars E and D by both PCR-based RFLP analysis and VS-2 sequencing. This discrepancy is most likely due to a double infection, which will, in most cases, result in the preferential amplification of 1 strain in the *omp1* PCR and PCR-based sequencing methods; in the TaqMan and Rotorgene assays, only LGV strains can be amplified. Whether this outbreak is partially technically driven must be assessed in the future by retrospectively investigating the presence of these LGV infections in men who have sex with men and the presence of the L2b strain in the past, since at present only LGV infections from 2003 to 2005 have been investigated.
